# Near Real-Time Biomass Burning PM2.5 Emission Estimation to Support Environmental Health Risk Management in Northern Thailand Using FINNv2.5

**DOI:** 10.3390/toxics14010084

**Published:** 2026-01-17

**Authors:** Chakrit Chotamonsak, Punnathorn Thanadolmethaphorn, Duangnapha Lapyai, Soottida Chimla

**Affiliations:** 1 Department of Geography, Faculty of Social Sciences, Chiang Mai University, Chiang Mai 50200, Thailand; soottida_chim@cmu.ac.th; 2 Environmental Science Research Center, Faculty of Science, Chiang Mai University, Chiang Mai 50200, Thailand; 3 Office of Strategy Management, Chiang Mai University, Chiang Mai 50200, Thailand; punnathorn.t@cmu.ac.th; 4 Office of Research Administration, Chiang Mai University, Chiang Mai 50200, Thailand; duangnapha.lapyai@cmu.ac.th

**Keywords:** biomass burning, PM2.5 emissions, near-real-time emission estimation, FINNv2.5, environmental health risk, Northern Thailand

## Abstract

Northern Thailand experiences recurrent seasonal haze driven by biomass burning (BB), which results in hazardous PM2.5 exposure and elevated environmental health risks. To address the need for timely and spatially resolved emission information, this study developed and evaluated an operational near-real-time (NRT) biomass-burning PM2.5 emission estimation system using the Fire INventory from NCAR version 2.5 (FINNv2.5). The objectives of this study are threefold: (1) to construct a high-resolution (≤1 km) NRT biomass-burning PM2.5 emission inventory for Northern Thailand; (2) to assess its temporal and spatial consistency with ground-based PM2.5 measurements and satellite fire observations; and (3) to examine its potential utility for informing environmental health risk management. The developed system captured short-lived, high-intensity burning episodes that defined the haze crisis, revealing a distinct peak period from late February to early April. Cumulative emissions from January to April 2024 exceeded 250,000 tons, dominated by Chiang Mai (25.8%) and Mae Hong Son (25.5%), which together contributed 51.3% of regional emissions. Strong correspondence with MODIS/VIIRS FRP (r = 0.79) confirmed the reliability of the NRT emission signal, while regression against observed PM2.5 concentrations indicated a substantial background burden (intercept = 40.41 μg m^−3^) and moderate explanatory power (R^2^ = 0.448), reflecting additional meteorological and transboundary influences. Translating these relationships into operational metrics, an Emission Control Threshold of 1518 tons day^−1^ was derived to guide targeted burn permitting and reduce population exposure during peak-risk periods. This NRT biomass-burning PM2.5 emission estimation framework offers timely emissions information that may support decision makers in environmental health risk management, including the development of early warnings, adaptive burn-permit strategies, and more coordinated responses across Northern Thailand.

## 1. Introduction

Biomass burning is the dominant source of fine particulate matter (PM2.5) and seasonal haze in Northern Thailand and across mainland Southeast Asia, with well-documented health, environmental and economic impacts. Numerous studies have indicated that agricultural residue burning and forest fires during the dry season are the primary contributors to regional PM2.5 pollution [1,2]. In Chiang Mai, biomass burning contributes to nearly 49% of the annual PM2.5, and more than 70% during April, while Bangkok also experiences notable increases during intense fire episodes [3,4,5,6]. Transboundary smoke originating from Myanmar and Laos further amplified PM2.5 levels in Northern Thailand [3,5,7]. Exposure to fire-related PM2.5 is strongly associated with respiratory and cardiovascular diseases, premature mortality, and disproportionate impacts on low-income and vulnerable populations [8,9,10]. Across Southeast Asia, fire-related PM2.5 has been linked to tens of thousands of premature deaths annually and economic losses exceeding USD 23 billion during severe haze years [8,9,10,11].

Biomass-burning haze in Northern Thailand follows a strongly seasonal pattern, typically peaking from February to April, when meteorological conditions, such as persistent temperature inversions, weak winds, and stagnant boundary layers, promote extreme smoke accumulation and long-range transport [3,5,12,13]. These environmental conditions highlight the urgent need for coordinated environmental health risk management on multiple scales. Although Thailand has implemented strict “zero-burning” policies, temporary no-burn periods, and other regulatory measures, enforcement challenges remain. Reactive approaches that rely on hotspot counts or observed PM2.5 often lag behind the actual burning activity, resulting in delayed interventions and critical PM2.5 spikes [6,14,15]. Strict enforcement has achieved short-term reductions of up to 50% in Chiang Mai; however, the haze season has lengthened from two to three months as farmers shift burning activities around policy windows rather than eliminating them [14]. These outcomes underscore the structural socioeconomic constraints, limited enforcement capacity in remote uplands, and scarcity of affordable alternatives to residue burning [14,16,17,18].

Recent scholarship advocates for proactive and integrated fire management strategies that combine real-time monitoring technologies, targeted outreach, financial incentives for alternative residue management, and participatory governance frameworks [16,17,19]. Digital burn registration platforms and public–private partnerships, including those linked to crop-processing industries, show considerable promise but remain limited in application [16]. Central to these emerging strategies is the availability of accurate, near-real-time biomass-burning emission inventories that can support timely environmental health risk assessments and evidence-based decision-making.

The Fire INventory from NCAR version 2.5 (FINNv2.5) represents a major advancement in this direction, producing global biomass-burning emission estimates at ~375 m daily resolution using improved MODIS and VIIRS active-fire detections, updated land-cover datasets, and revised emission factors [20]. Parallel global fire-emission products, such as GFED, GFAS, and QFED, leverage fire radiative power (FRP) to provide near-real-time emission estimates [21,22,23]. These inventories are increasingly used for air quality forecasting, exposure assessment, and emission control policy development [21,23,24]. However, uncertainties remain substantial, particularly in regions with small or short-lived fires and frequent cloud cover. Cross-inventory discrepancies as high as a factor of six for CO and 8.7 for organic carbon have been reported owing to differences in burned-area detection algorithms and emission factors [20,22,25,26]. For Southeast Asia, several studies have recommended integrating multiple datasets and refining emission factors to better reflect regional vegetation characteristics [25,27].

Despite these advances, the operational adoption of FINNv2.5 for real-time governance and environmental health risk management in Southeast Asia remains limited. Existing applications are predominantly research-focused, emphasizing retrospective analyses rather than embedding daily emissions into actionable decision thresholds for proactive interventions [28,29]. Incorporating near-real-time emissions into fire management protocols, such as conditional burn permits or early warning systems, has strong potential to mitigate extreme haze episodes and reduce population exposure [7,18,26,27,30]. However, no published studies have demonstrated a fully operational FINNv2.5-based system integrated within Thailand’s fire-management infrastructure [28,29].

Recent regional initiatives provide strong evidence for the feasibility of high-resolution NRT PM2.5 emission estimation for Northern Thailand using FINNv2.5. Localized implementations combining MODIS/VIIRS detections, land cover, and vegetation-specific emission factors have produced daily ~1 km inventories that show strong spatial and temporal agreements with observed PM2.5 and satellite fire detections [18,30,31]. Advances in machine learning techniques have further enhanced predictive performance by incorporating meteorological variables and additional satellite observations [28,30,32].

Accordingly, this study developed and evaluated an operational near-real-time biomass-burning PM2.5 emission estimation system for Northern Thailand using FINNv2.5 as the core framework. The specific objectives are as follows:

1. We constructed a high-resolution (≤1 km) NRT biomass-burning PM2.5 emission inventory for Northern Thailand.
2. Validate system outputs against ground-based PM2.5 measurements and satellite hotspot/FRP observations data.
3. Demonstrate the system’s decision-support potential for proactive, evidence-based environmental health risk management to prevent severe haze.

## 2. Materials and Methods

### 2.1. Study Area

**Figure 1:**
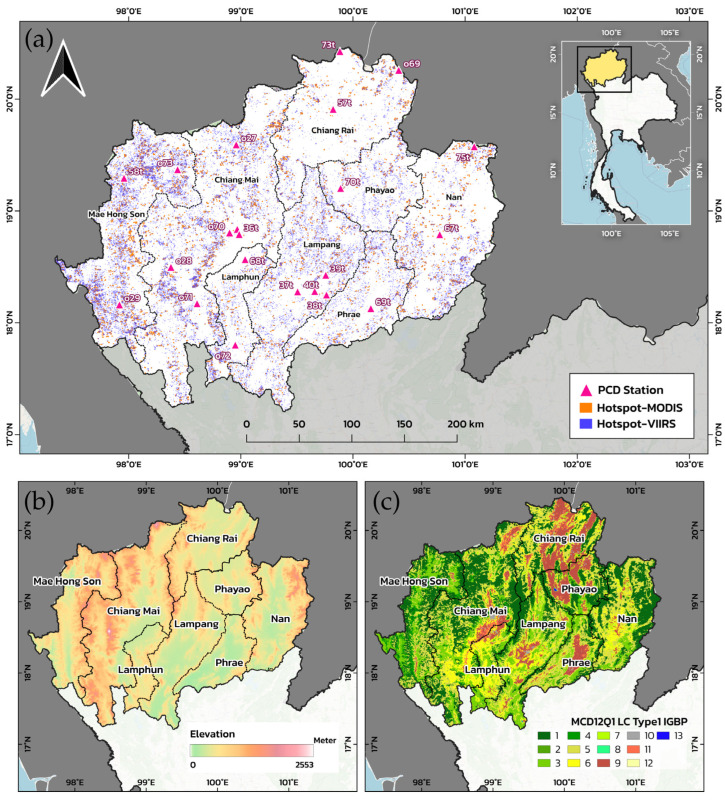
Study area in Northern Thailand. (**a**) Spatial distribution of fire hotspots detected by MODIS and VIIRS (Suomi-NPP), together with Pollution Control Department (PCD) monitoring stations. (**b**) Elevation of Northern Thailand derived from the Shuttle Radar Topography Mission (SRTM). (**c**) Land-cover classification based on the MODIS MCD12Q1 Collection 1 Type-1 IGBP scheme, including: (1) Evergreen broadleaf forests, (2) Deciduous needleleaf forests, (3) Deciduous broadleaf forests, (4) Mixed forests, (5) Woody savannas, (6) Savannas, (7) Grasslands, (8) Permanent wetlands, (9) Croplands, (10) Urban and built-up areas, (11) Natural vegetation mosaics, (12) Barren lands, and (13) Water bodies.

This study focuses on the eight northern provinces of Thailand—Chiang Mai, Chiang Rai, Lamphun, Lampang, Phrae, Nan, Phayao, and Mae Hong Son—located between approximately 15–21° N and 97–101° E (Figure 1). This region is widely recognized as one of the most fire-prone landscapes in mainland Southeast Asia, characterized by extensive upland forests and agricultural mosaics that are highly susceptible to biomass burning during the dry season [3,4,5]. The terrain comprises rugged mountain ranges interspersed with intermontane basins, creating complex local meteorology and spatially heterogeneous fire behaviors. Elevations range from ~200 m in the valley plains to over 2500 m in the northern highlands.

The climate of Northern Thailand is governed by a pronounced monsoon cycle, with a wet season from May to October and a dry season from November to April. The mean annual rainfall is typically 1100–1400 mm, but prolonged dry-season droughts, accumulated crop residues, and low relative humidity create highly favorable conditions for open burning. Fire activity peaks between February and April, coinciding with the pre-monsoon period, when atmospheric stagnation, persistent temperature inversions, and weak winds trap smoke near the surface and drive extreme PM2.5 concentrations [6,7].

The study area encompasses diverse land cover types, including dry dipterocarp, mixed deciduous, and evergreen forests, together with extensive agricultural land, making Northern Thailand a representative testbed for near-real-time biomass-burning emission estimations. The complex mountain–valley topography strongly modulates smoke transport, and during the dry season, restricted dispersion combined with nocturnal inversions promotes the accumulation of PM2.5 in deep valleys and basins [8]. Chronic exposure to these pollution episodes has been directly linked to an increased burden of respiratory diseases, COPD, and lung cancer among local populations [9,15,33], underscoring the importance of environmental health risk management. Land use is a primary driver of regional biomass-burning emissions and the associated seasonal air quality crisis. The landscape is dominated by approximately 62% forest and 31% agricultural land, providing the structural conditions for recurrent fire activity. Biomass burning occurs predominantly during the dry season (January–April) and is fueled by two main sources: agricultural-residue burning and forest fires. This seasonal concentration and source diversity allow for the assessment of emission variability across different combustion regimes within a consistent climatic window.

Agricultural burning contributes substantially to seasonal emissions, particularly through the widespread burning of crop residues from maize monocultures for rapid land preparation and traditional rotational shifting cultivation in upland areas [16,34]. Maize residue burning is highly synchronized in time and often peaks sharply during March–April, coinciding with the most severe haze conditions in Northern Thailand.

Forest fires are the second major emission source, driven by extensive and fire-prone forest cover. Mixed deciduous and deciduous dipterocarp forests shed large quantities of leaf litter during the dry season, generating abundant and highly combustible surface fuel. Rising temperatures, declining rainfall, and prolonged dry spells further enhance fuel availability and ignition potential, intensifying the fire severity and spatial extent [35]. Together, these characteristics make the region well-suited for evaluating emission estimation performance across varying fuel types, fire behaviors, and spatial scales.

**Figure 2:**
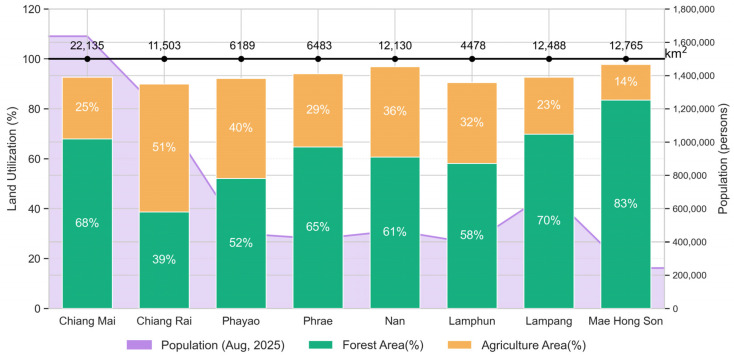
Land-use composition (forest and agricultural areas) and population distribution across eight provinces in Northern Thailand, illustrating the spatial overlap between the dominant land-use types and population exposure.

**Figure 3:**
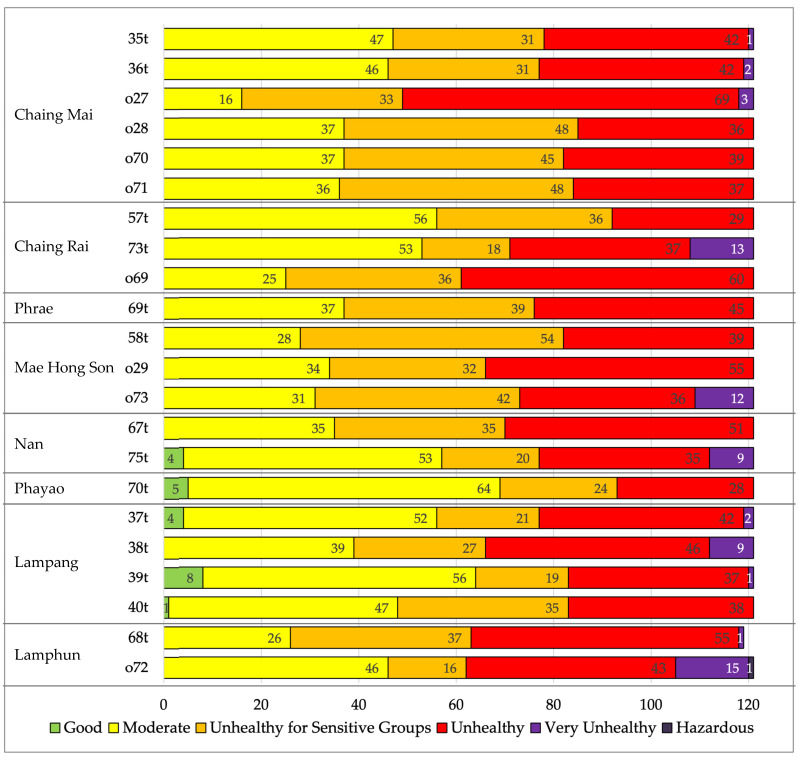
Number of days by PM2.5 air-quality category (US AQI) at monitoring stations across Northern Thailand from January to April 2024, illustrating the frequency of unhealthy and hazardous pollution levels by province. Detailed information for each monitoring station is provided in Table A1.

Figure 2 summarizes land-use characteristics and population density in 8 provinces, highlighting the high proportion of forest and agricultural land, which are contributing factors to repeated biomass burning and associated environmental health risks. The overlap of high population densities in intermontane basins and intense burning zones creates significant exposure. Epidemiological evidence from Northern Thailand highlights that health risks are increased not only by peak concentrations but also by the duration of exposure during haze periods. Studies in Chiang Mai showed that seasonal haze significantly impaired the quality of life and lung function in patients with chronic obstructive pulmonary disease (COPD), with effects persisting throughout periods of high pollution [33]. Furthermore, recent analyses have found a correlation between increasing air pollution levels and the prevalence of acute pulmonary embolism, indicating serious cardiovascular effects, in addition to respiratory distress [36]. In Chiang Rai, health risk assessments have revealed that heavy metal components in PM2.5 during haze periods pose significant carcinogenic and non-carcinogenic risks to local communities [37]. Moreover, vulnerable populations, such as primary school children, exhibit patterns of limited lung function that are directly linked to PM2.5 exposure, underscoring a long-term threat to public health in the region [38]. These studies show that eight provinces in upper Northern Thailand are at risk of high pollution. The majority of the study days were within the range of having an impact on health, as shown in Figure 3, according to the US AQI level criteria.

### 2.2. Data Sources

Developing a near-real-time PM2.5 emission estimation system for Northern Thailand requires the integration of multiple complementary datasets (Table 1) that capture fire activity, landscape characteristics, and air quality conditions. Core fire information was obtained from NASA’s Fire Information for Resource Management System (FIRMS) using the MODIS MCD14DL active-fire product [39] and the higher-resolution VIIRS VNP14IMGTDL product [40]. MODIS provides global coverage at a 1 km spatial resolution, whereas VIIRS detects fires at a 375 m resolution, enabling the detection of smaller, fragmented burns typical of mountainous agricultural landscapes. For each fire pixel, key attributes, including location, detection time, Fire Radiative Power (FRP), and confidence level, were collected for 2019–2025. Detections with confidence <80% were excluded, and overlapping MODIS–VIIRS detections were screened within a 500 m buffer to remove duplicates while retaining the highest-confidence record. Land cover information was derived from the MODIS MCD12Q1 classification [41] at a 500 m resolution, providing detailed vegetation classes, including evergreen and deciduous forest types, mixed forest, woody savanna, grassland, cropland, wetland, and urban areas. Because emission factors and fuel loads depend strongly on vegetation, the MCD12Q1 classes were cross-checked against land-use maps from Thailand’s Land Development Department to ensure consistency with local biophysical conditions and agricultural practices. Ground-based air quality observations were used as independent references to evaluate the temporal behavior of the emission estimates. Hourly PM2.5 measurements from nine stations in the Pollution Control Department (PCD) network were aggregated to daily means for January–April 2024. Only stations with ≥80% data completeness were retained to ensure robust validation. Administrative boundaries (province and district polygons) were obtained from the Department of Provincial Administration (DOPA) and standardized to the WGS84 geographic coordinate system (EPSG:4326), ensuring the consistent alignment of satellite detections, land cover maps, emission fields, and ground observations within the geospatial workflow.

### 2.3. Fire Data Detection Processing

**Figure 4:**
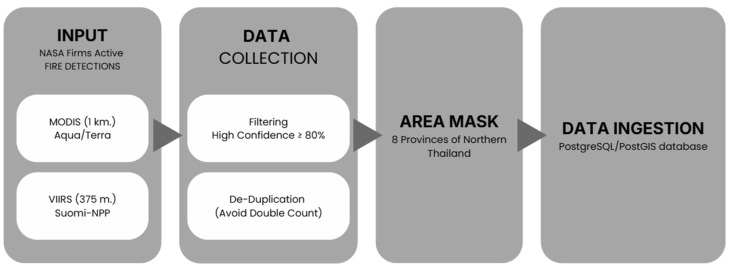
Preprocessing workflow for satellite active-fire detections used in near-real-time PM2.5 emission estimation. MODIS and VIIRS fire detections were filtered by confidence, de-duplicated, spatially masked to Northern Thailand, and ingested into a PostgreSQL/PostGIS database.

Processing satellite fire detections is a central step in preparing reliable inputs for the FINNv2.5-based emissions system [20]. Because raw MODIS and VIIRS hotspots differ in spatial resolution, detection sensitivity, and overpass timing, a dedicated preprocessing workflow (Figure 4) was designed to transform the daily FIRMS data into a harmonized, high-quality fire dataset suitable for near-real-time emission calculations.

The workflow begins with the daily ingestion of MODIS (1 km) and VIIRS (375 m) active fire detections. Although both sensors observe the same landscape, VIIRS generally detects more small and low-intensity agricultural burns, whereas MODIS provides a longer measurement record. To ensure that the combined dataset reflected true fire activity, a multi-step quality control procedure was applied. First, a confidence filter was implemented, retaining only detections with a confidence level > 80%. This threshold, based on the contextual fire detection algorithm of Giglio et al. (2003) [39], effectively removes thermal anomalies unrelated to open burning (e.g., industrial heat sources, volcanoes, or sun glint), thereby reducing commission errors. Second, a spatiotemporal deduplication filter was used to address overlapping detections from the two sensors. MODIS and VIIRS may detect the same fire at different times or resolutions; therefore, detections within a 500 m radius and a 24 h window were merged into a single fire event, following established hotspot fusion methods [42]. This step prevented double counting and ensured that each fire contributed only once to the emission calculations. Finally, the cleaned hotspot dataset was geographically masked to retain only the fire activity within the eight northern provinces. This ensured that the emission system focused on regional biomass burning while excluding detections outside the study domain, except where transboundary effects were explicitly examined. Through this structured pipeline, raw FIRMS detections were transformed into a consistent, quality-assured, and region-specific fire dataset (Figure 4).

**Figure 5:**
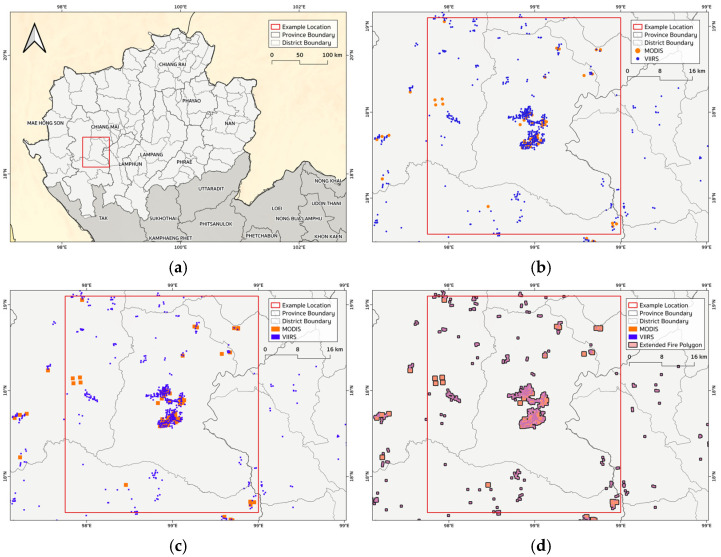
Processing of satellite fire detections for burned-area estimation: (**a**) example location in Northern Thailand, (**b**) MODIS and VIIRS fire detections, (**c**) pixel-based fire polygons, and (**d**) merged burned-area patches used in the FINNv2.5 emission calculation.

Following initial quality control, the core processing stage converted the cleaned fire detections into an effective burned area, a required input to the FINNv2.5 emission equation (Figure 5). As shown for the Jom Thong District in Chiang Mai, each hotspot was first associated with its sensor-specific pixel size: 1 km^2^ for MODIS and 0.14 km^2^ (375 m × 375 m) for VIIRS [40]. Each detection was then converted into a square polygon, yielding preliminary burned-area patches. These polygons were merged and clustered based on spatial overlap and proximity to represent contiguous fire events and avoid artificially fragmented burned areas in the analysis.

This geometry-based transformation was performed in a PostgreSQL/PostGIS environment. The resulting burned-area polygons were intersected with the MODIS MCD12Q1 land cover layer to assign each fire patch an appropriate vegetation class. This step was essential because the land cover type determines the biomass loading, combustion completeness, and emission factors used in the FINNv2.5 calculation [20]. The final processed dataset comprised a daily, spatially explicit effective burned area linked to land cover attributes, ready for use in the near-real-time PM2.5 emission workflow.

### 2.4. FINNv2.5 Framework and Regional Adaptation

**Figure 6:**
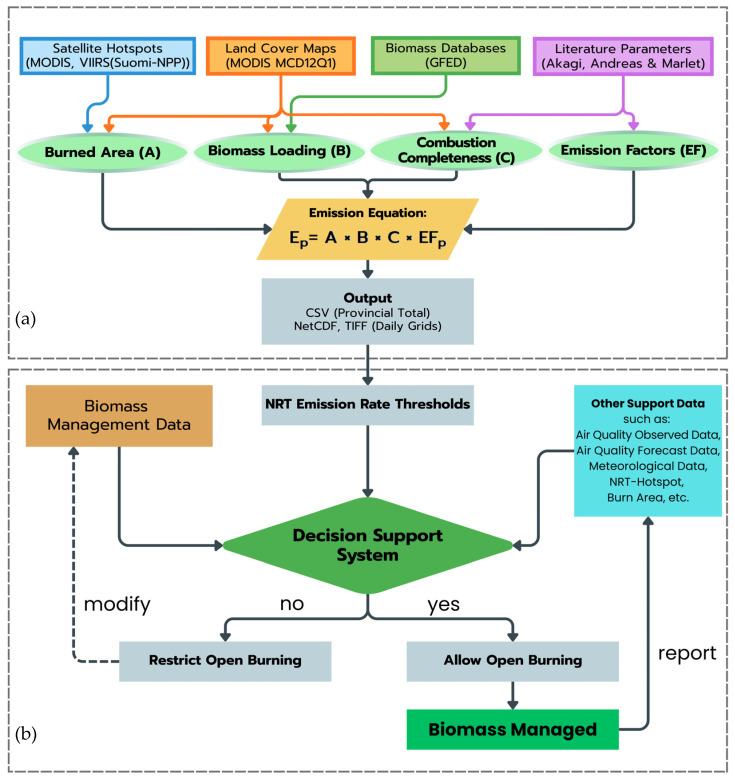
Conceptual workflow of the operational near-real-time PM2.5 emission estimation system based on the FINNv2.5 framework for environmental health risk management. (**a**) Satellite-derived fire activity, land cover, biomass loading, combustion completeness, and emission factors were integrated to generate daily PM2.5 emission fields. (**b**) Near-real-time emission rates are combined with air quality observations, forecasts, and meteorological information within a decision-support system to support risk-informed regulation of open burning and mitigation of population exposure.

The FINNv2.5 framework [20] formed the foundation of the near-real-time biomass-burning emissions system (Figure 6). Although originally designed as a global product, FINNv2.5 was adapted to reflect the ecological conditions and burning practices of Northern Thailand, where mixed deciduous forests, dipterocarp woodlands, and maize-dominated agricultural landscapes create diverse fuel environments. The system links satellite-detected fire activity with fuel availability and burning efficiency to produce daily PM2.5 emissions at a resolution suitable for both scientific modeling and operational environmental health risk management.

At the core of FINNv2.5 is the assumption that each detected fire pixel contains information about (i) effective burned area (*A*), (ii) biomass loading (*B*), (iii) combustion completeness (*C*), and (iv) PM2.5 emission factor (*EF*). These components are combined as

(1) $$E_{{\mathrm{PM}}_{2.5}}=A\times B\times C\times EF$$

where $E_{{\mathrm{PM}}_{2.5}}$ is the emitted PM2.5 mass.

The effective burned area (*A*) was derived from the cleaned MODIS and VIIRS detections described above, which were merged and spatially aggregated to represent contiguous fire clusters. VIIRS, with its smaller footprint, is particularly important for capturing small agricultural burns in valleys and highlands. Biomass loading (*B*) was assigned according to the underlying land cover class (MCD12Q1). Default FINNv2.5 values were used as the baseline, representing the average dry fuel mass per hectare, while acknowledging that they mask local variability—for example, between dipterocarp forests and highland maize fields [43,44]. This is recognized as a source of structural uncertainties. Combustion completeness (*C*) is also linked to land cover. Dry-season deciduous forests, which accumulate thick leaf litter, generally burn more completely than moist evergreen forests, whereas agricultural residues may burn rapidly but incompletely. Values were drawn from the literature [45,46] and FINNv2.5 defaults, applied with consideration of regional fuel conditions. The emission factor (*EF*) converts the burned fuel into PM2.5, expressed as the mass of PM2.5 per kg of dry matter consumed. *EF* values were adopted from global compilations [47,48] and assigned according to vegetation type and burning conditions.

Daily high-resolution PM2.5 emission fields and provincial summaries were generated as outputs, which are suitable for use in chemical-transport modeling and direct interpretation in environmental health risk management. Beyond the model input, the emission estimates were embedded within a decision-support logic (Figure 6b): a daily emission threshold calibrated against the observed PM2.5 is used as a screening tool. When a burn request is submitted, the system evaluates the proposed activity using multiple information layers, including forecast meteorology, air quality predictions, recent hotspots, and burned area records. If projected emissions are likely to push concentrations toward hazardous levels, the system flags the request for restriction; under more favorable conditions, burning may be authorized as “managed biomass burning”.

This feedback loop allows observed fires, whether permitted or not, to refine thresholds and improve subsequent decisions. Over time, the NRT emission system has functioned as an adaptive governance tool that balances public health protection with the practical needs of farmers and land managers. This process creates a feedback loop. Every fire that occurs—authorized or otherwise—contributes new information. Confirmed burned areas and hotspot detections were fed back into the system, gradually refining the threshold and improving decision-making. Consequently, the NRT emission system has evolved into a living, adaptive governance tool, capable of balancing public health concerns with the practical needs of farmers and land managers. It offers not only emissions but also a transparent, evidence-based protocol for determining when, where, and under which conditions burning should proceed.

### 2.5. System Architecture and Workflow

**Figure 7:**
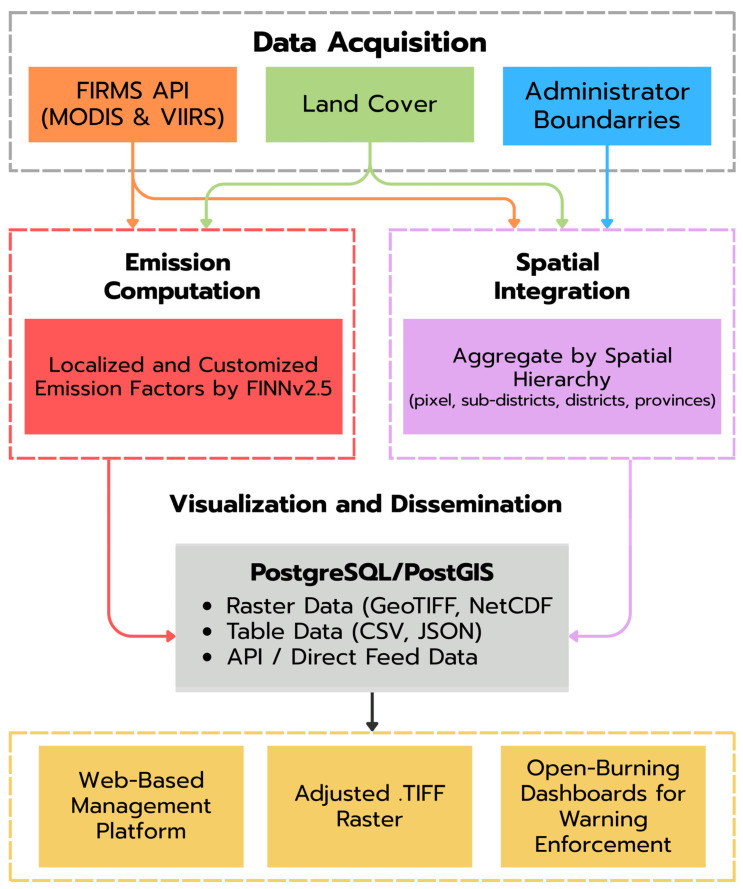
Workflow of the near-real-time PM2.5 emission rate analysis system. Satellite active-fire detections from the FIRMS API (MODIS and VIIRS), together with land-cover data and administrative boundaries, are ingested for emission computation using localized FINNv2.5 parameters. Emission outputs are spatially aggregated across multiple administrative levels and stored in a PostgreSQL/PostGIS database before being disseminated through raster products, tabular data, APIs, and web-based dashboards to support open-burning management and environmental health risk mitigation.

The near-real-time emission system was implemented as a fully automated data pipeline that transformed raw satellite fire detections into operational information for biomass-burning management (Figure 7). The workflow comprises four integrated components: (i) data acquisition, (ii) emission computation, (iii) spatial integration, and (iv) visualization and dissemination of the results.

In the data acquisition module, daily MODIS and VIIRS active fire detections were retrieved automatically using the NASA FIRMS API. These dynamic inputs were combined with static spatial layers, including land cover (MCD12Q1), administrative boundaries, and auxiliary geospatial datasets defining the analytical domain.

In the emission-computation module, the adapted FINNv2.5 algorithm was applied to each valid fire pixel. The effective burned area was derived from sensor-specific footprints, followed by the assignment of biomass loading, combustion completeness, and emission factors based on land cover. Localized parameter tables calibrated for Northern Thailand enable daily PM2.5 emission maps at a ~1 km resolution. The computations were implemented in Python 3.10 using optimized geospatial libraries to ensure rapid processing.

The spatial integration module then ingests the emission outputs into a PostgreSQL/PostGIS spatial database. Emissions were organized hierarchically by pixel, subdistrict, district, and province, allowing for multiscale queries and temporal aggregation. This structure supports the real-time tracking of cumulative emissions, hotspot density, and spatial clustering of fire activity, which are key inputs for environmental health risk assessment and management.

In the final visualization and dissemination module, the outputs are converted into user-ready products. Daily GeoTIFF rasters, NetCDF files, and tabular summaries (CSV/JSON) were generated and made available for air quality forecasting models and web-based management dashboards. These products allow provincial agencies and national platforms to access spatially detailed emissions data and integrate them into burn-permit monitoring, early warning systems, and enforcement mechanisms.

The entire workflow was orchestrated using cron-based scheduling on a Linux server, enabling fully automated daily updates. The modular architecture supports regional operations and provides a scalable foundation for national or ASEAN-level open burning management systems.

### 2.6. Validation and Performance Evaluation

Validation of the near-real-time emission system focused on how well modeled PM2.5 emissions captured actual fire activity and surface air-quality conditions. Three complementary dimensions were considered: temporal correlation, spatial consistency, and statistical performance.

For temporal evaluation, daily emissions from the adapted FINNv2.5 algorithm were compared with daily surface PM2.5 concentrations from the PCD network. Pearson correlation coefficients (r) were computed between the emission time series and observed PM2.5 at each station to assess whether emission peaks aligned with pollution episodes and to what extent the system reproduced day-to-day variability.

Spatial consistency was evaluated by overlaying daily PM2.5 emission maps with hotspot density and FRP distributions. Agreement between high-emission zones and clusters of hotspots/FRP served as an indicator that the system correctly translated localized fire activity into regional emission patterns across land-cover types, elevation bands, and fire regimes.

To quantify performance more formally, we calculated the correlation coefficient (r), coefficient of determination (R^2^), and parameters of regression models relating emissions to concentrations.

### 2.7. Implementation Environment

The operational emission system was deployed on an Ubuntu 22.04 LTS server that was configured for high-throughput geospatial processing. The platform, equipped with a 32-core CPU and 128 GB of RAM, provides sufficient capacity for daily MODIS/VNIRS ingestion, geospatial transformation, and regional-scale emission computation.

The software stack combines modern open-source tools for automation, spatial computation and data integration. Python 3.10 was used as the primary scripting language for data ingestion, preprocessing, emission computation, and output generation. The core libraries included NumPy v1.24.4, Pandas v2.0.3, Rasterio v1.3.11, GDAL v3.4.3, Matplotlib v3.7.5, and SQLAlchemy v2.0.37. Spatial data were stored in a PostgreSQL 15 database with PostGIS 3.3, enabling hierarchical storage (pixel → subdistrict → district → province) and high-performance spatial querying and aggregation. QGIS 3.28 was used for visualization, map preparation, and the manual verification of spatial outputs.

Automation is handled by daily cron-based scheduling, ensuring that all system components—from satellite data retrieval through emission computation to product dissemination—run without manual intervention. The architecture supports API-level communication with external platforms, including burn-permit management systems, early warning dashboards, and provincial or national air quality management frameworks.

Overall, this implementation provides a robust, scalable, and replicable platform for generating near-real-time PM2.5 emission estimates. By bridging satellite fire detection with structured geospatial computation and automated data delivery, the system underpins proactive air quality and environmental health governance in Northern Thailand and can be adapted to other biomass-burning regions across Southeast Asia.

## 3. Results

### 3.1. Seasonal Dynamics of PM2.5 Emissions

**Figure 8:**
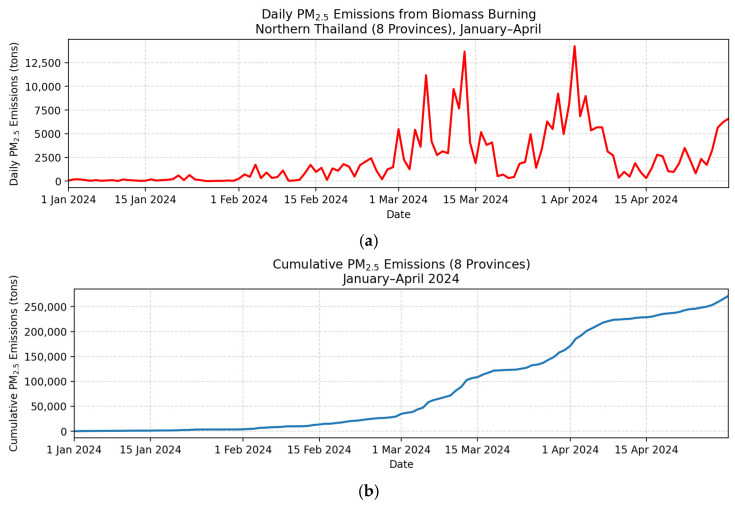
Near-real-time daily (**a**) and cumulative (**b**) biomass-burning PM2.5 emissions in Northern Thailand (eight provinces) from January to April 2024, estimated using FINNv2.5.

The operational near-real-time emission system clearly captured the seasonal evolution of biomass-burning PM2.5 emissions in Northern Thailand (Figure 8). As illustrated in Figure 8a, the daily emissions exhibited a distinct and highly volatile pattern aligned with the region’s open-burning cycle. During January and early February, emissions remained relatively low—typically below 2500 tons day^−1^—reflecting limited burning activity in the early dry season.

A pronounced transition occurred in late February, when emissions began to increase rapidly. Throughout March and early April, the system detected repeated high-intensity burning episodes, including extreme events exceeding 13,660 tons day^−1^ (13 March 2024) and peaking at 14,233 tons day^−1^ (2 April 2024). This cluster of explosive emission pulses indicates that a substantial proportion of the annual biomass-burning pollution burden occurs within a short and predictable crisis window. These bursts are closely associated with synchronized post-harvest maize residue burning and widespread upland forest fires in the region.

The cumulative emissions curve (Figure 8b) reinforces the magnitude and temporal concentration of pollution load. While cumulative totals increased slowly during January–February, they rose sharply beginning in early March, mirroring the abrupt increases in the daily emissions. By the end of April, the cumulative BB-PM2.5 emissions exceeded 250,000 tons, underscoring the extraordinary scale of pollutant release during the peak-burning season.

The environmental and health implications of this condensed emission period are significant. The release of large pollutant loads within only a few weeks dramatically elevates population exposure and intensifies environmental health risks, especially for vulnerable groups, such as children, the elderly, and individuals with respiratory diseases. High-frequency, high-magnitude emission spikes also strain local health systems and impose economic burdens on tourism, labor productivity and regional healthcare expenditures.

These findings highlight a central insight: the Northern Thailand haze crisis is not a gradual seasonal phenomenon but a sharply defined short-term emission surge. Effective environmental health risk management therefore requires (i) rapid monitoring of daily emission escalation, (ii) targeted, time-sensitive intervention during the March–April crisis window, and (iii) coordinated cross-border responses to address concurrent fire activity in neighboring countries.

The near-real-time FINNv2.5-based system provides the temporal precision necessary to anticipate these emission peaks, enabling proactive mitigation rather than delayed and reactive responses.

### 3.2. Spatial Distribution and Cumulative Emissions

**Figure 9:**
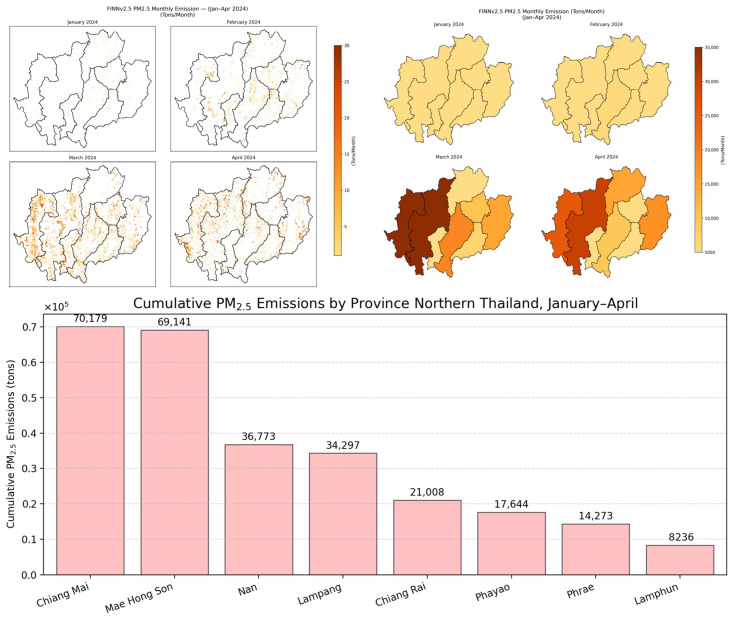
Spatial distribution and cumulative biomass-burning PM2.5 emissions across eight provinces of Northern Thailand from January to April 2024, estimated using FINNv2.5. Monthly maps show the seasonal intensification of emissions, while the bar chart highlights the cumulative provincial contributions, dominated by Chiang Mai and Mae Hong Son.

The spatial distribution of biomass-burning PM2.5 emissions exhibited pronounced heterogeneity across Northern Thailand during the January–April fire season (Figure 9). At the beginning of the haze season in January, the emissions were relatively modest and spatially diffuse. However, by February, the spatial extent and intensity of emissions expanded across nearly all provinces, reflecting the onset of widespread residue burning and early dry-season forest fires. Emissions reached their maximum in March, when the western and central highlands exhibited the most severe fire activity. Although April still recorded elevated emissions, a gradual decline emerged as pre-monsoon rainfall began to suppress ignition and fire spread.

This seasonal intensification is clearly reflected in the monthly provincial totals. Mae Hong Son and Chiang Mai consistently emerged as the dominant sources of emissions. In March, Mae Hong Son generated ~40,346 tons month^−1^ and Chiang Mai ~34,821 tons month^−1^; both declined somewhat in April to 25,431 tons month^−1^ and 30,210 tons month^−1^, respectively. Other provinces, particularly Nan and Lampang, contributed substantially but at lower magnitudes, whereas Lamphun recorded the smallest emissions (average ~2059 tons month^−1^). Collectively, these findings indicate a concentrated emission pulse during the late winter and early spring months, with the epicenter located along the mountainous, western corridor.

Cumulative emissions revealed an even more striking pattern of provincial disparities. Over the full four-month period, Chiang Mai (70,179 tons) and Mae Hong Son (69,141 tons) were the largest contributors, jointly accounting for a substantial proportion of regional PM2.5 burden. Their elevated contributions reflect not only extensive forest cover but also the prevalence of fire-prone landscapes and intensive residue-burning practices in March and April. These spatially concentrated emission burdens provide critical evidence for designing targeted regulatory strategies, as they identify the provinces where intervention will yield the most substantial reductions in population exposure.

Provinces such as Nan (36,773 tons) and Lampang (34,297 tons) formed a secondary tier of contributors, whereas Lamphun (8236 tons) consistently exhibited the lowest cumulative emissions values. This stratified provincial ranking highlights the necessity of differentiated policy responses—high-intensity enforcement and fuel management in the most fire-active provinces, paired with supportive but less resource-intensive measures in provinces with minimal burning activity.

**Figure 10:**
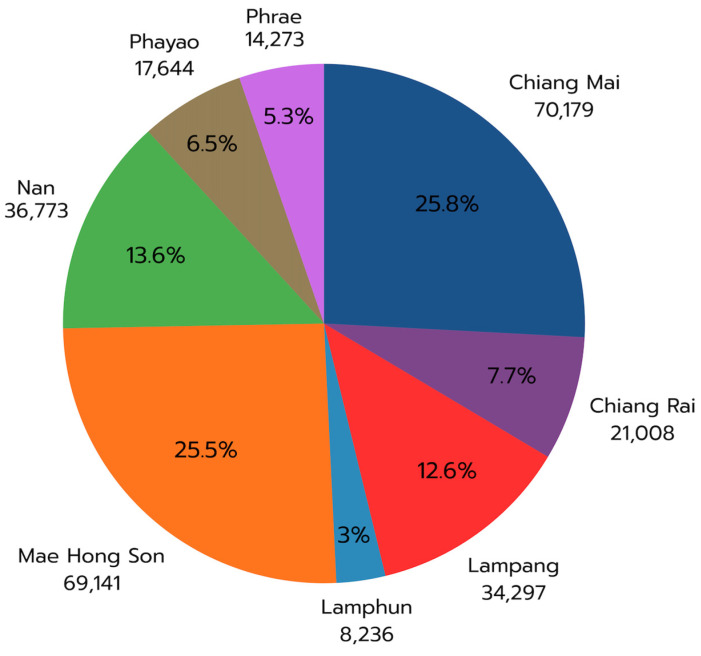
Provincial percentage contributions to cumulative biomass-burning PM2.5 emissions in Northern Thailand (January–April 2024), highlighting the dominant contributions of Chiang Mai and Mae Hong Son.

The percentage contribution analysis (Figure 10) further emphasizes the extreme concentration of emissions: Chiang Mai (25.8%) and Mae Hong Son (25.5%) produced 51.3% of all biomass-burning PM2.5 emissions in Northern Thailand. The remaining six provinces contributed the other half, led by Nan (13.6%) and Lampang (12.6%), again underscoring the geographic asymmetry of the pollution sources.

**Figure 11:**
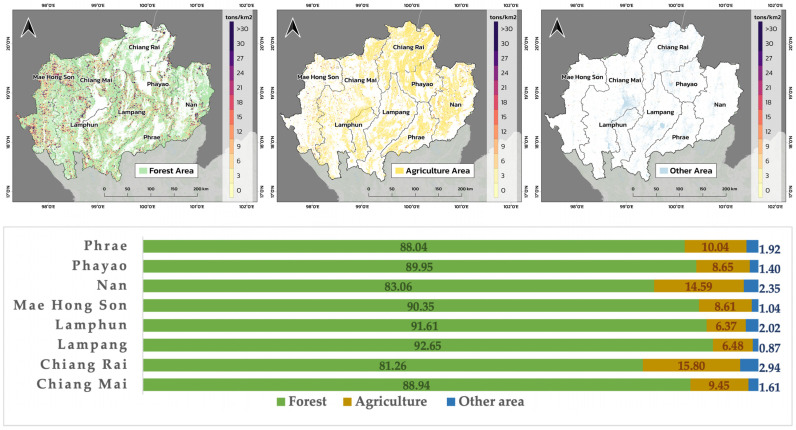
Spatial distribution and provincial contributions of biomass-burning PM2.5 emissions by land-use type across Northern Thailand. The upper panels show emission density maps (tons km^−2^) for forest, agricultural, and other land-use categories, while the lower panel summarizes the proportional contributions of each land-use type to total emissions in eight provinces. Emissions were estimated using FINNv2.5.

Land-use-based emission analysis (Figure 11) revealed that all eight provinces contained >80% forest cover, with Lampang having the highest (92.65%) and Chiang Rai the lowest (81.26%). Chiang Rai also exhibited the highest agricultural share (15.80%) followed by Nan (14.59%). These land-use structures shape the emission dynamics of provinces.

Forest-based emissions differed markedly across provinces (Table 2). Although Chiang Mai and Mae Hong Son have similar total forest-derived PM2.5 emissions (~62,400 tons), their emission intensity diverges substantially when normalized by forest area. Mae Hong Son has the highest forest emission density (5.86 tons km^−2^), indicating more intense biomass consumption per unit area than Chiang Mai (4.15 tons km^−2^). Phayao emerged as an unexpected hotspot, with a forest emission density nearly as high (4.93 tons km^−2^), despite its smaller forest area. In contrast, Lamphun and Chiang Rai showed lower forest emission densities (2.90 and 3.84 tons km^−2^, respectively), indicating that even when fires do occur, fuel consumption and fire intensity are lower than in the western border provinces.

Agricultural emission patterns revealed even greater provincial contrasts. Chiang Rai—despite having the largest agricultural area (5905 km^2^)—shows the lowest agricultural emission intensity (0.56 tons km^−2^), indicating either well-controlled residue burning or crops with lower residue loads. Mae Hong Son, however, presents an opposite pattern: with only 1821 km^2^ of cropland, it produced the highest agricultural emission intensity (3.27 tons km^−2^), nearly six times that of Chiang Rai and more than twice that of Chiang Mai (1.21 tons km^−2^) and Nan (1.22 tons km^−2^). This disproportionate intensity likely reflects the prevalence of upland shifting cultivation and slope agriculture, where extensive fire use is integral to land clearing and the disposal of residues. These results highlight that uniform agricultural haze policies are insufficient and that Mae Hong Son specifically requires interventions tailored to highland farming systems.

The significant variation in emission magnitude and intensity across provinces indicates the need for asymmetric and targeted policy interventions. Concentrating resources on Chiang Mai and Mae Hong Son through strengthened enforcement, agricultural waste management, and economic incentives would yield the greatest reductions in regional PM2.5 exposure. Secondary provinces, such as Nan and Lampang, should also receive prioritized support, albeit with resource allocation proportional to their emission contributions. Therefore, effective haze mitigation depends on the strategic prioritization of high-emitting provinces to maximize the impact of limited government capacity during the March–April crisis period.

The spatial distribution patterns of emissions were highly consistent with hotspot detections, reinforcing the reliability of the FINNv2.5-based system in capturing the geographic extent of daily burning activity. The next section evaluates the temporal correspondence between the estimated emissions and ground-based PM2.5 observations and satellite fire detections to further confirm the system performance.

### 3.3. Consistency with Fire Activity Observations

**Figure 12:**
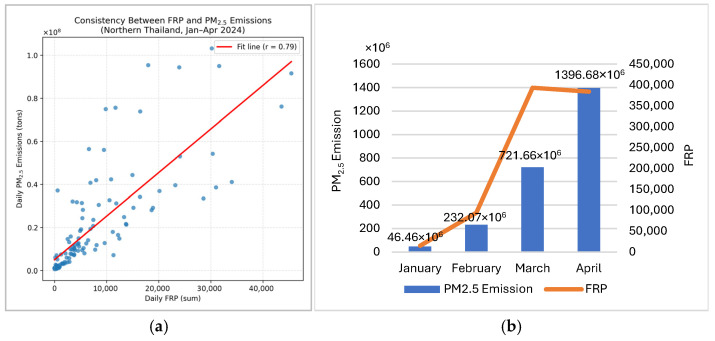
Temporal consistency between MODIS/VIIRS Fire Radiative Power (FRP) and FINNv2.5 near-real-time PM2.5 emission estimates in Northern Thailand (January–April 2024): (**a**) daily relationship between FRP and PM2.5 emissions and (**b**) monthly aggregated PM2.5 emissions and FRP, illustrating a coherent seasonal escalation.

The temporal pattern of PM2.5 emissions produced by the near-real-time FINNv2.5 system closely mirrored the fire radiative power (FRP) detected by MODIS and VIIRS across Northern Thailand from January to April 2024 (Figure 12). This agreement at both daily and monthly timescales confirms that the emission model effectively captures the timing, frequency, and intensity of biomass-burning events that drive the region’s dry-season haze.

At the daily scale (Figure 12a), a strong positive correlation was observed between the FINNv2.5 emissions and satellite-detected FRP. A scatter plot analysis of the daily FRP and PM2.5 emissions from January to April 2024 yielded a Pearson correlation coefficient of r = 0.79, indicating that increases in fire intensity were directly associated with corresponding increases in estimated PM2.5 emissions. This strong co-variability highlights the reliability of FRP as a near-real-time proxy for monitoring fire severity and associated pollutant loads, particularly in regions where ground-based observations remain sparse.

A broader seasonal coherence was observed when comparing the monthly aggregates (Figure 12b). Monthly PM2.5 emissions increased exponentially from January to their peak in April, reaching approximately 1.4 × 10^9^ units. This trend closely aligned with the seasonal trajectory of FRP, which rose sharply beginning in February and remained elevated throughout March and April. The persistence of high emissions in April, despite the stabilization or slight declining, suggests additional contributions from meteorological factors, such as stagnant conditions and reduced atmospheric dispersion, as well as smoke accumulation over consecutive burning days. These factors collectively exacerbate population exposure and reinforce the need for proactive environmental health risk management during late-season peaks.

**Figure 13:**
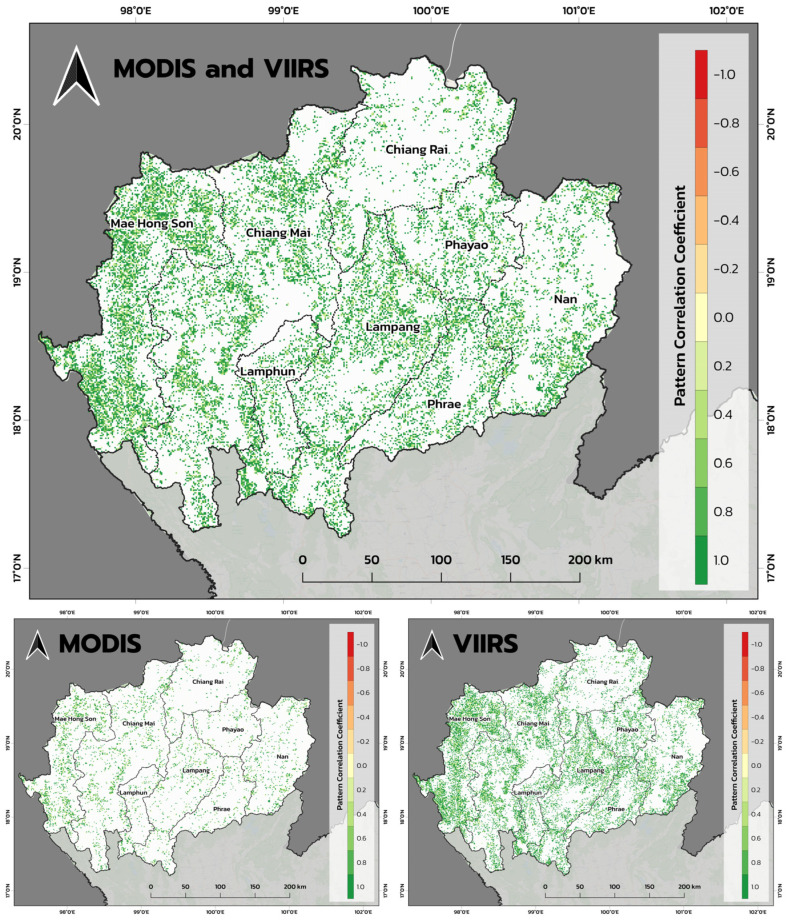
Spatial distribution of correlation coefficients between daily MODIS/VIIRS Fire Radiative Power (FRP) and FINNv2.5 PM2.5 emission rates across Northern Thailand, illustrating a strong spatial consistency in major biomass-burning areas.

Spatial consistency further validated the accuracy of the emission system. As shown in Figure 13, the spatial correlations between the daily FINNv2.5 emissions and MODIS/VIIRS FRP were consistently high across the region, with most provinces exhibiting Pearson correlation coefficients exceeding 0.8. Agreement was particularly strong in major burning provinces, such as Mae Hong Son, Chiang Mai, Nan, Lampang, and Phrae, indicating that the system effectively reproduced the spatial structure of fire activity across diverse ecological and land-use contexts.

Lower correlations in a few subregions corresponded to areas with complex terrain or persistent cloud cover, conditions that reduce the sensitivity of satellite-based FRP retrievals and occasionally obscure small or short-lived fires. These discrepancies reflect limitations in satellite detection, rather than deficiencies in the emission model.

Overall, the temporal and spatial coherence between FINNv2.5 emissions and observed fire activity demonstrated that the system provides a robust and operationally reliable representation of daily biomass-burning dynamics in Northern Thailand. This reliability is essential not only for fire-risk monitoring and biomass-fuel management but also for driving downstream air quality forecasting models and supporting environmental health risk mitigation during the critical March–April haze period.

### 3.4. Application to Decision Support

**Figure 14:**
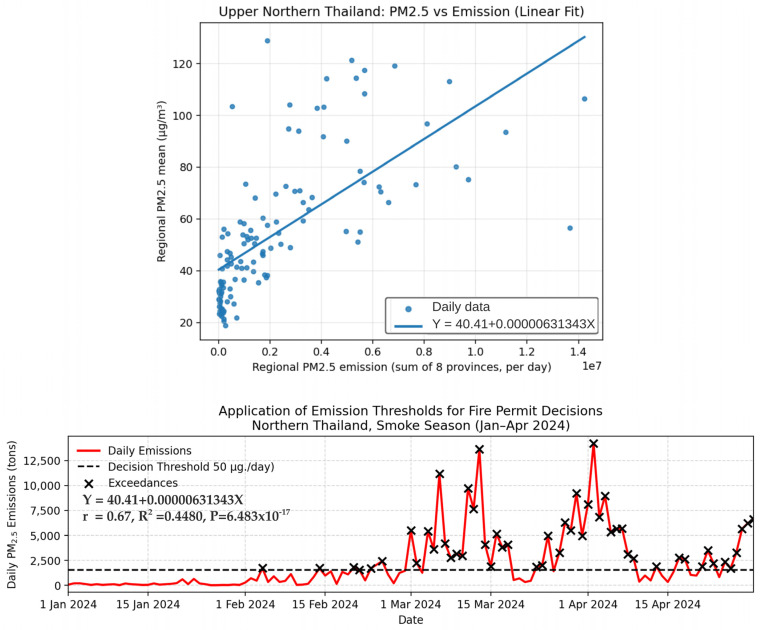
Application of daily PM2.5 emission thresholds for fire permit regulation during the smoke season in Northern Thailand (January–April 2024). The top panel shows the relationship between regional mean PM2.5 and daily emissions, while the bottom panel illustrates daily emissions with the threshold of 1518 tons day^−1^; exceedance days indicate periods when permit restrictions may be required.

To translate near-real-time emissions into actionable policy guidance, we established a quantitative link between daily FINNv2.5-derived biomass-burning emissions and surface PM2.5 concentrations across eight provinces in Northern Thailand. A bivariate linear regression model was employed, where the daily PM2.5 concentrations (*Y*, μg m^−3^) were regressed against the daily emission rates (*X*, tons) (Figure 14). This approach provides a statistically defensible mechanism for determining when fire activity is likely to elevate PM2.5 levels beyond the health-relevant thresholds. The resulting regression model is as follows:

(2) Y=40.41+0.00000631343X

Regression Equation (2) yields several insights that are central to air quality governance. The model exhibited a highly significant statistical relationship (*p *= 6.483 × 10^−17^) and moderate correlation (*r *= 0.67), indicating that days with higher emissions were consistently associated with elevated surface PM2.5. The intercept (α = 40.41 μg m^−3^) is particularly noteworthy, suggesting that even in the hypothetical absence of all biomass-burning emissions, Northern Thailand experiences baseline concentrations that exceed the national 24 h PM2.5 standard of 37.5 μg m^−3^. This elevated baseline reflects a combination of urban emissions, regional transport, and persistent atmospheric stagnation during the dry season.

The coefficient of determination (*R*^2^ = 0.448) indicates that approximately 45% of the day-to-day PM2.5 variability can be attributed to biomass burning emissions. The unexplained variance likely results from meteorological processes (e.g., boundary layer dynamics, wind fields, and humidity) and transboundary smoke inflows. Nevertheless, the strength of the emission–concentration relationship is sufficient to support operational decision-making.

### 3.5. Deriving an Operational Emission Control Threshold

Because the legal PM2.5 standard is routinely exceeded even under zero-burning conditions, a more pragmatic threshold is required for real-world management. Therefore, we adopted 50 μg m^−3^ as an actionable decision-support benchmark, consistent with regional warning levels and widely used public health advisory thresholds.

By substituting *Y *= 50 μg m^−3^ into Equation (2), we obtained the maximum permissible daily emission rate: *X* = 1518 tons day^−1^. This value is proposed as the Emission Control Threshold—the maximum daily biomass-burning emission load that should not be exceeded to maintain PM2.5 at or below the selected operational limits.

Figure 14 illustrates the application of this threshold in regulating fire-permit issuance. During the peak burning season (mid-March to early April 2024), actual emissions frequently exceeded 10,000 tons day^−1^, breaching the threshold for several days. These exceedances highlight both the severity of uncontrolled burning and the necessity of a dynamic response system rather than static seasonal bans on burning.

### 3.6. Adaptive, Threshold-Based Burn Management

The introduction of a threshold-based permitting system represents a major advance over traditional blanket-burning prohibitions. Historically, static bans have produced several unintended consequences, including the following:

- displacement of burning into narrower, more intense windows immediately before or after official restrictions
- increased illegal burning in remote highland areas, and
- reduced cooperation from rural communities whose agricultural cycles depend on controlled fire use

By linking burn authorizations to real-time emission conditions, the threshold-based framework enables a more flexible and socially acceptable approach to prescribed burning. This allows controlled burning during periods with naturally low emissions or favorable dispersion (e.g., January–February, post-April), while restricting activities during high-risk episodes. This adaptive model:

- encourages compliance by aligning rules with environmental reality
- reduces incentives for illegal burning
- strengthens trust between authorities and communities
- shifts behavior to minimize emissions during the most health-critical weeks of the year

This integrated approach delivers maximum public health benefits while acknowledging the livelihood constraints of the rural population of Northern Thailand.

### 3.7. Integration with the Fire Management Decision Support System

**Figure 15:**
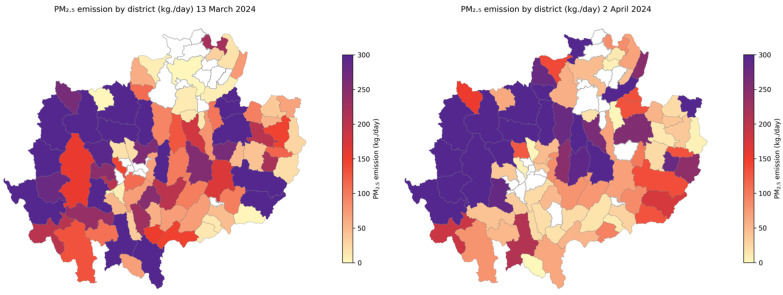
Example of District-level PM2.5 emissions derived from the near-real-time FINNv2.5 system for two representative peak burning days: 13 March 2024 (**left**) and 2 April 2024 (**right**).

Emission outputs from the FINNv2.5 system were ingested into the operational Fire Management Decision Support System (Fire-D) platform, where they augmented station observations, satellite data, and meteorological forecasts. Sub-district-level (tambon) emission summaries (Figure 15) support the following:

- early warning and anticipatory decision-making
- cross-agency coordination
- dynamic burn-permit evaluation
- fuel-management prioritization

To maximize accessibility and ensure broad adoption, near-real-time (NRT) emission products are disseminated through multiple Fire-D channels.

- Web platform: https://fire-d.com (accessed on 12 September 2025)
- iOS application: https://apps.apple.com/th/app/fired/id1567748564 (accessed on 12 September 2025)
- Android app: https://play.google.com/store/apps/details?id=com.rcces.fired (accessed on 12 September 2025)
- LINE Official Account: https://line.me/R/ti/p/@426typfk (accessed on 12 September 2025)

These distribution pathways allow provincial officers, district authorities, community fire response teams, and national agencies to continuously monitor emerging risk conditions and respond proactively to emission spikes.

By embedding scientifically derived thresholds into everyday decision workflows, the combined FINNv2.5–Fire-D system establishes a robust foundation for evidence-based, health-oriented biomass-burning governance across Northern Thailand.

Figure 15 illustrates how district-level emission estimates can be operationalized in a decision-support context. The spatial patterns observed on 13 March and 2 April 2024 demonstrate the system’s ability to highlight localized emission hotspots during peak burning days, enabling authorities to identify high-risk districts and strategically allocate fire suppression resources. By providing near-real-time, spatially explicit emission fields, the system allows decision makers to monitor evolving conditions, anticipate potential exceedances of air quality thresholds, and implement timely burn-permit restrictions. These district-level insights form a critical operational layer within the Fire-D platform, bridging scientific emission modeling with practical governance needs and creating a foundation for responsive, evidence-based environmental health risk management in Northern Thailand.

## 4. Discussion

### 4.1. Quantifying the Crisis: The Value of NRT Emission Data

Deploying FINNv2.5 in a near-real-time (NRT) configuration provides a high-resolution quantitative lens on the dynamics of biomass burning (BB) PM2.5 in Northern Thailand. Compared with coarser monthly products such as GFED, the daily temporal resolution and ~1 km spatial detail of FINNv2.5 are critically important for operational air-quality management and short-term forecasting [20,23]. NRT outputs capture the abrupt, short-lived emission surges that characterize severe haze episodes, which are often blurred or completely missing in monthly scale inventories [22]. Capturing these daily fluctuations is critical. Recent epidemiological evidence from Northern Thailand indicates that health outcomes, particularly in patients with Chronic Obstructive Pulmonary Disease (COPD), are driven by the duration of exposure rather than sporadic peaks alone [33]. By resolving emissions at a daily scale, the NRT system identifies consecutive high-emission days that lead to cumulative inflammatory responses and acute exacerbations, providing a level of health risk assessment that monthly inventories cannot support.

In this study, NRT emissions revealed a sharply defined crisis window extending from late February to early April, during which cumulative emissions exceeded 250,000 tons. The pronounced spatial asymmetry of emissions, where Chiang Mai (25.8%) and Mae Hong Son (25.5%) together contributed 51.3% of the regional total (Figure 8, Figure 9, Figure 10 and Figure 11), underscores how a small number of provinces disproportionately drive the period of peak pollution. These daily, spatially explicit datasets form an actionable evidence base for provincial haze committees, national regulators, and transboundary policy negotiations [8,11,29].

### 4.2. Reliability of Estimates and Model Limitations

The strong temporal agreement between FINNv2.5-derived emissions and satellite-based Fire Radiative Power (FRP) demonstrates the capability of the adapted system to capture the frequency, intensity, and seasonal evolution of biomass burning. A correlation coefficient of r = 0.79 (Figure 12) confirmed that variations in combustion intensity were effectively translated into daily emission estimates, indicating robust performance for near-real-time monitoring of fire activity [39,40].

In contrast, a comparison with ground-based PM2.5 observations reveals inherent limitations. The moderate explanatory power (R^2^ = 0.448) indicates that emissions alone account for approximately 45% of the daily PM2.5 variability, with the remaining variance governed by meteorological and transportation processes. Factors such as atmospheric stagnation, temperature inversions, shallow boundary layer heights, and weak ventilation during the dry season strongly modulate pollutant accumulation and dispersion [3,5,27]. The elevated regression intercept (40.41 µg m^−3^) further reflects a substantial baseline PM2.5 burden associated with persistent regional pollution, transboundary smoke inflows, and local anthropogenic sources [7,49].

Northern Thailand is periodically influenced by cross-border smoke transport from neighboring regions, which elevates the background PM2.5 concentrations, independent of local biomass burning activity. These transboundary contributions partially explain the non-zero intercept and limited ability of emissions alone to predict surface concentrations. Together, these results highlight that near-real-time emission estimates represent a necessary but insufficient condition for PM2.5 prediction, underscoring the importance of coupling emission information with meteorological fields and regional transport processes for a comprehensive air quality assessment.

Several structural uncertainties inherent to FINNv2.5 must be acknowledged. Hotspot-based burned area estimation may underestimate emissions from small, short-lived, and cloud-obscured fires [42,50]. In addition, FINNv2.5 relies on globally averaged biomass loading (B) and emission factors (*EF*), which may not fully capture the fuel characteristics of Northern Thailand, particularly post-harvest maize residues and deciduous dipterocarp leaf litter [43,44,47,48]. Therefore, emission estimates are sensitive to uncertainties in *B* and *EF*, potentially introducing systematic bias in regions dominated by specific land-use practices.

Satellite fire detection is subject to limitations, including reduced sensitivity under persistent cloud cover and diminished detection of low-intensity or short-duration fires. These gaps may lead to a localized underestimation of the burned area and associated PM2.5 emissions, particularly from fragmented agricultural landscapes. While such limitations do not affect the temporal consistency of regional emission patterns, they introduce uncertainty at fine spatial and daily scales. Importantly, these constraints do not undermine the utility of FINNv2.5 for operational near-real-time applications but rather highlight opportunities for region-specific parameterization and adaptive calibration to improve quantitative accuracy [20,24].

### 4.3. Policy Application: From Emission Data to Decision Support

Despite inherent uncertainties, the observed emission–concentration relationship provides substantial operational value for air quality governance. Baseline PM2.5 concentrations in Northern Thailand frequently exceed the national 24 h standard of 37.5 µg m^−3^ even under minimal burning conditions, largely due to persistent regional pollution and transboundary inflows [14,15]. Consequently, a higher operational threshold is required to translate near-real-time emission information into meaningful decision-support signals.

In this study, a PM2.5 threshold of 50 µg m^−3^ was adopted as a pragmatic action point reflecting conditions under which acute health risks demonstrably increase, and governance intervention becomes necessary. Epidemiological evidence has linked short-term PM2.5 exposure above this level to adverse respiratory and cardiovascular outcomes, including restrictive lung function in children and increased incidence of acute pulmonary embolism [36,38]. Using this threshold, the regression model yielded a regional Emission Control Threshold of 1518 tons day^−1^ (Figure 14), representing the estimated atmospheric carrying capacity beyond which prolonged pollution episodes are likely to occur.

Consistent exceedances above this threshold coincided with elevated daily emission loads and stagnant meteorological conditions, confirming its suitability as a practical trigger for adaptive burn permitting and short-term risk mitigation. During the peak haze period (March–April), daily emissions frequently exceeded 10,000 tons day^−1^, far surpassing the threshold and justifying strict temporary restrictions. In contrast, emissions during January and February generally remained below the threshold, supporting conditional burn permits that facilitate early-season fuel reduction.

Rather than functioning as a strict regulatory limit, the threshold serves as a decision-support indicator that converts near-real-time emission estimates into actionable environmental governance signals. This adaptive, threshold-based approach improves upon blanket seasonal bans, which have been shown to induce unintended behavioral responses such as compressed burning windows and increased illegal activity [16,51,52,53]. By aligning intervention timing with real-time atmospheric capacity, the framework enables more flexible, context-sensitive, and socially acceptable fire management while enhancing public health protection.

From a sustainability perspective, the proposed framework directly contributes to multiple United Nations Sustainable Development Goals (SDGs). By reducing population exposure to harmful particulate matter, it supports SDG 3 (Good Health and Well-Being), while its application to urban–rural haze mitigation and adaptive decision-making aligns with SDG 11 (Sustainable Cities and Communities). The integration of satellite-based monitoring with operational emission estimation advances SDG 13 (Climate Action) through strengthened early warning and response capacity, and land-use-specific emission attribution supports improved fire management and ecosystem protection under SDG 15 (Life on Land).

Although demonstrated for Northern Thailand, the framework is transferable to other biomass-burning-prone regions, particularly across Southeast Asia and the Global South. Its reliance on globally available satellite products, standardized emission inventories, and scalable spatial databases enables application in regions with limited ground-based monitoring. As such, the proposed approach offers a replicable model for integrating air quality science into adaptive environmental governance and cooperative transboundary haze mitigation strategies.

### 4.4. Broader Implications: Local Hotspots vs. Transboundary Haze

The pronounced spatial heterogeneity in emissions has major implications for regional haze management in Northern Thailand. The dominance of Chiang Mai and Mae Hong Son reflects their landscape characteristics, fuel availability, and proximity to transboundary smoke transport corridors [5,34,35]. High-resolution NRT emissions data help distinguish locally driven pollution days from those dominated by transboundary inflows, supporting differentiated local, national, and regional responses.

For domestic policy, these insights enable targeted enforcement, tailored fuel management programs, and geographically prioritized public health interventions.

Furthermore, differentiating between local sources is essential for assessing toxicity risks. Research in Chiang Rai suggests that PM2.5 during haze episodes contains specific heavy metal components that pose a carcinogenic risk [37]. By identifying the precise location and land-use type of active fires (e.g., agricultural vs. forest), the NRT system can help health authorities anticipate not only the mass of PM2.5, but also its chemical toxicity, enabling more specific public health warnings.

At the regional scale, the transparency provided by NRT emissions strengthens evidence-based negotiations under the ASEAN Agreement on Transboundary Haze Pollution, improving accountability, enhancing cooperation, and facilitating coordinated mitigation strategies [52,53,54].

### 4.5. Future Research Directions

Two major research avenues have strong potential to advance the accuracy, predictive power, and operational relevance of near-real-time emission systems in Southeast Asia:

#### 4.5.1. Integration of NRT Emissions with Chemical Transport Models (CTMs)

Coupling FINNv2.5 outputs with models such as WRF-Chem would enable proactive air quality forecasting that incorporates meteorology and atmospheric chemistry. This integration would allow for multi-day prediction of downwind PM2.5 concentrations, supporting early warnings, public health advisories, and optimized resource deployment [27,55]. CTM coupling also allows for the assessment of plume-rise behavior, boundary layer impacts, and cross-border transport, factors that are not explicitly represented in emission inventories.

#### 4.5.2. Regionalization of Biomass Loading (*B*) and Emission Factors (*EF*)

Local studies show that biomass loading and *EF* values for key fuels, such as maize residues and dipterocarp leaf litter, differ substantially from global defaults [56]. These parameters directly influence the emission equation [20].

The use of global averages may introduce systematic bias. Region-specific empirical measurements can significantly reduce structural uncertainty and improve quantitative accuracy [43,44,47,48].

An adaptive calibration framework that periodically updates parameters using hotspot density, burn scar products, and receptor observations would further improve performance and account for interannual variability in fire behavior [23,24].

Together, these future directions will strengthen the role of NRT emission systems in science-based fire governance, public health protection, and regional air quality prediction.

## 5. Conclusions

This study demonstrates the feasibility and operational value of near-real-time (NRT) PM2.5 emission estimation system for Northern Thailand based on the FINNv2.5 framework. By integrating daily MODIS and VIIRS fire detections with land cover information and regionally adapted geospatial processing, the system captures the short, intense burning episodes that characterize the region’s seasonal haze crisis. The NRT emission record revealed a sharply defined peak from late February to early April, with cumulative emissions exceeding 250,000 tons, more than half of which originated from only two provinces: Chiang Mai and Mae Hong Son (51.3%). The strong temporal correspondence with Fire Radiative Power (FRP) from MODIS and VIIRS (r = 0.79) confirms the robustness of the emission estimates for situational awareness and early warning.

Linking emissions to surface PM2.5 concentrations through a simple regression model highlights both the promise and limitations of emissions-based decision support. The high baseline PM2.5 concentration (40.41 µg m^−3^) and moderate explanatory capability (R^2^ = 0.448) underscore the combined influence of atmospheric conditions and transboundary smoke. Nonetheless, the derivation of an operational Emission Control Threshold of 1518 tons day^−1^ offers a practical, quantitative mechanism for adaptive burn permitting. During crisis periods—when emissions frequently exceed 10,000 tons day^−1^—strict restrictions are warranted, whereas lower-risk windows (e.g., January–February) allow for regulated, conditional burning that balances environmental protection with the livelihood needs of rural communities.

The proposed NRT biomass burning PM2.5 emission estimation system provides three immediate operational benefits.

1. Daily high-resolution emission fields are suitable for integration with chemical transport models, enabling predictive PM2.5 forecasting and proactive public-health advisories.
2. Transparent, sub-provincial evidence supports targeted enforcement, resource prioritization, and coordinated interagency responses.
3. A shared empirical foundation for domestic policy design and transboundary engagement under the ASEAN Agreement on Transboundary Haze Pollution.

Future development should prioritize (i) the full coupling of NRT emissions with chemical transport models such as WRF-Chem and (ii) the regionalization of biomass-loading and emission-factor parameters for dominant fuels, particularly maize residues and deciduous dipterocarp leaf litter. These improvements will enhance predictive accuracy, strengthen early warning capabilities, and improve the effectiveness and credibility of haze mitigation strategies across Northern Thailand and the broader Mekong subregion.

## Figures and Tables

**Table 1:** Summary of all datasets, including spatial and temporal resolution, data sources, and pre-processing steps.

Dataset	Spatial Resolution	Temporal Resolution	Data Sources	Pre-Processing Steps
Active Fire Detection (Fire Attributes)	MODIS: 1 km	Daily	NASA FIRMS (Fire Information for Resource Management System) products:	Excluded pixels with detection confidence < 80%.
	VIIRS: 375 m	MODIS (MCD14DL) and VIIRS (VNP14IMGTDL).	Removed duplicate detections between MODIS and VIIRS via 500 m spatial overlay.	
Land Cover Classification (Used for emission factors and fuel-load)	500 m	Annual (Implied by MCD12Q1 product)	MODIS MCD12Q1 product.	Cross-checked with national land-use maps from Thailand’s Land Development Department (LDD) for local consistency.
Ground-based Air Quality Data (PM2.5)	Point data (9 stations)	Hourly (Aggregated to Daily)	Pollution Control Department (PCD) monitoring network.	Aggregated hourly to daily averages. Included only stations with data completeness > 80%.
Administrative Boundaries	Vector/Polygon data	Static	Thailand’s Department of Provincial Administration (DOPA).	Projected onto the WGS84 coordinate system (EPSG: 4326).

**Table 2:** Comparative analysis of forest and agricultural areas, derived emissions, and emission intensity per unit area in the Upper North provinces.

Province		Chiang Mai	Chiang Rai	Lampang	Lamphun	Mae Hong Son	Nan	Phayao	Phrae
Total	Area (km^2^)	22,135	11,503	12,488	4478	12,765	12,130	6189	6783
	PM2.5 Emissions (tons)	70,179	21,008	34,297	8236	69,141	36,773	17,644	14,273
	Emission Density (tons/km^2^)	3.17	1.83	2.75	1.84	5.42	3.03	2.85	2.10
Forest Area	Area (km^2^)	15,037	4441	8721	2600	10,655	7359	3221	4793
	Emissions (tons)	62,421	17,072	31,775	7545	62,469	30,544	15,870	12,566
	Emission Intensity (tons/km^2^)	4.15	3.84	3.64	2.90	5.86	4.15	4.93	2.62
Agricultural Area	Area (km^2^)	5475	5905	2844	2844	1821	4395	2485	1905
	Emissions (tons)	6631	3320	2223	525	5956	5366	1527	1433
	Emission Intensity (tons/km^2^)	1.21	0.56	0.78	0.18	3.27	1.22	0.61	0.75

## Data Availability

All data generated or analyzed in this study are included in this article. Additional information or access to the derived datasets is available from the corresponding author upon reasonable request.

## Appendix A

**Table A1:** List of air quality monitoring stations included in this study.

Station Code	Station Name	Province
35t	Chiang Mai Provincial Government Center	Chiang Mai
36t	Yuparaj Wittayalai School	Chiang Mai
o27	Muang Na Subdistrict Municipality Office	Chiang Mai
o28	Thep Ratana Vejchanukul Hospital, Mae Chaem	Chiang Mai
o70	Phuping Palace	Chiang Mai
o71	Provincial Electricity Authority, Hod District Branch	Chiang Mai
57t	Chiang Rai Provincial Office of Natural Resources and Environment	Chiang Rai
73t	Mae Sai Public Health Office	Chiang Rai
o69	Wiang Subdistrict Municipality Disaster Prevention and Mitigation Center	Chiang Rai
69t	Phrae Provincial Meteorological Office	Phrae
58t	Mae Hong Son Provincial Office of Natural Resources and Environment	Mae Hong Son
o29	Mae Sariang Public Health Office	Mae Hong Son
o73	Pai Airport	Mae Hong Son
67t	Nan Municipality Office	Nan
75t	Chalermprakiat Hospital	Nan
70t	Phayao Provincial Stadium	Phayao
37t	Lampang Meteorological Station	Lampang
38t	Ban Sop Paad Subdistrict Health Promotion Hospital	Lampang
39t	Tha Si Subdistrict Health Promotion Hospital	Lampang
40t	Mae Moh Provincial Waterworks Authority	Lampang
68t	Lamphun Provincial Meteorological Office	Lamphun
o72	Li District Office	Lamphun
